# The erratic antibiotic susceptibility patterns of bacterial pathogens causing urinary tract infections

**DOI:** 10.17179/excli2015-207

**Published:** 2015-08-04

**Authors:** Iftkhar Ahmed, Muhammad Sajed, Aneesa Sultan, Iram Murtaza, Sohail Yousaf, Bushra Maqsood, Petr Vanhara, Mariam Anees

**Affiliations:** 1 Department of Biochemistry, Faculty of Biological Sciences, Quaid-i-Azam University, Islamabad, Pakistan; 2 Department of Environmental Sciences, Faculty of Biological Sciences, Quaid-i-Azam University, Islamabad, Pakistan; 3 Department of Medicine Unit 2, Holy Family Hospital, Rawalpindi, Pakistan; 4 Department of Histology and Embryology, Faculty of Medicine, Masaryk University, Brno, Czech Republic; 5 Institute of Health and Management Sciences, Shaheed Zulfiqar Ali Bhutto Medical University, Islamabad, Pakistan

**Keywords:** Escherichia coli, Klebsiella pneumoniae, ESBL, antibiotic susceptibility

## Abstract

Increasing trend of antibiotic resistance and expression of Extended Spectrum Beta Lactamases (ESBLs) are serious threats for public health as they render the treatment ineffective. Present study was designed to elucidate the antibiotic-susceptibility patterns of ESBL and non-ESBL producing *E. coli *and *K. pneumoniae *causing urinary tract infections so that the ineffective antibiotics could be removed from the line of treatment. The bacterial isolates obtained from the urine of patients visiting a tertiary health care facility were cultured for strain identification using API20E. Antimicrobial susceptibility and ESBL detection were done by Kirby-bauer diffusion technique. Almost 53.4 % isolates of *E. coli* and 24.5 % isolates of *K. pneumoniae* were found to be ESBL producers. The ESBL producing bacteria were found to be more resistant towards various antibiotics. The most effective drugs against *E. coli* ESBL isolates were imipenem (99.54 %), ampicillin-sulbactam (97.48 %), piperacillin-tazobactam (96.86 %), fosfomycin (94.51 %), amikacin (92.26 %) and nitrofurantoin (90.68 %). The most effective drugs against *K. pneumoniae* ESBL isolates were imipenem (97.62 %), piperacillin-tazobactam (95.35 %), ampicillin-sulbactam (90.48 %) and amikacin (88.37 %). The antibiotics having the highest resistance, particularly by the ESBL producers were amoxicillin clavulanic acid, sulphamethoxalzole/ trimethoprim, cefuroxime, cefpirome, ceftriaxone and ciprofloxacin. Most of the isolates showed multi drug resistance (MDR). High frequency of ESBL producing *E. coli* and *K. pneumoniae* were observed as compared to previous data. Penicillins, cephalosporins and some representatives of fluoroquinolones were least effective against the common UTIs and are recommended to be removed from the line of treatment.

## Introduction

Microbes causing urinary tract infections (UTIs) affect all age and gender groups unanimously. Clinical manifestations of UTIs vary greatly leading to severe morbidity and even mortality. Uropathogenic organisms are mostly human intestinal commensals. These organisms show virulent behavior upon transmission to clinically significant biological sites (Boye and Hansen, 2003[6]; Jonas et al.*,* 2004[14]). *Escherichia coli, *a facultative anaerobe and gram negative bacillus, has been reported as the most prevalent microbe followed by *Klebsiella pneumoniae *and other pathogens like *Staphylococcus, Proteus, Pseudomonas, Enterococcus*, and *Enterobacter *(Farrel et al., 2003[12]; Mirsoleymani et al., 2014[16]). In the past few years, an increasing trend in the antibiotic resitance imparted by *E. coli *and *K. pneumoniae *isolates has been observed. This worrisome shift in resistance is posing a greater therapeutic challenge (Sharma et al.*,* 2007[24]) as the treatment of UTIs is often impaired due to the resistance of leading pathogens to commonly used antimicrobial agents (Chakupurakal et al.*,* 2010[7]; Souli et al.*,* 2010[25]). One of the resistance mechanisms developed by *E. coli *and *K. pneumoniae *is the phenotypic expression of plasmid mediated genes encoding Extended Spectrum Beta Lactamases (ESBL). Most of the beta lactam antibiotics used in the treatment of clinical bacteriuria are rendered non-susceptible by these enzymes. The prevalence of ESBL producing strains is found to be higher than other resistant phenotypes proving them as threat for clinical management (Hyle et al.*,* 2005[13]). The malpractices leading to resistant attributes of microorganisms include substandard antibiotic quality, unhygienic environment responsible for transmission of resistant strains, inadequate surveillance and antibiotic administration by health professionals and misuse by patients (Okeke et al., 2005[18]). Treatment options have become limited due to the drastic increase in resistance against various classes of antibiotics including fluoroquinolones and all cephalosporin generations. This phenomenon can augment the severity of even a simple UTI (Shariff et al., 2013[23]). The antibiotic susceptibility of pathogens show high inter regional variation and is linked to the clinical practices of physicians and medication practices of patients along with evolutionary resistance which make certain pathogens more resistant over time. This study was undertaken to determine the antibiotic susceptibility patterns of ESBL and non-ESBL producing *E. coli* and *K. pneumoniae* in a tertiary care facility in Islamabad, Pakistan.

## Methodology

The samples were collected at Department of Microbiology, Pakistan Institute of Medical Sciences, Islamabad, Pakistan, from patients visiting the hospital with clinical symptoms of urinary tract infection. The study was ethically approved from the Institutional Review Board of Quaid-i-Azam University, Islamabad, Pakistan. Informed consent was obtained from the patients before including them in the study. The patients were guided to pass the midstream urine sample by un-touched, sterile technique in a wide-mouth screw capped sterile container. A total of 6254 urine samples were collected from males and females belonging to all age groups.

For the screening of bacterial infection, the urine samples were cultured on blood agar and MacConkey agar plates and the colony morphology was studied after 24 hours incubation at 37 °C. Colonies from agar plate isolates were smeared on glass slides and stained with Gram's stain using Preston's and Morrells modification (Cheesbrough, 2006[8]) to examine for Gram's reaction, colour, size, shape and distribution of the microrganisms. The strains were further tested for ESBL phenotye using disc diffusion method by applying panel of beta lactam drugs (cefuroxime, ceftriaxone, cefpirome and imipenem) followed by strain identification by Analytical Profile Index (API 20 E). Susceptibility of the isolates was determined against a panel of antibiotic discs including augmentin, cefuroxime, ceftriaxone, cefpirome, norfloxacin, ciprofloxacin, amikacin, gentamicin, sulfamethoxalzole-trimethoprim, nitrofurantoin, fosfomycin, imipenem, ampicillin-sulbactam, piperacillin-tazobactam. CLSI guidelines were used for susceptibility markers of each of the antibiotics applied to declare the antibiotic as resistant or sensitive.

Two-sided Student's t-tests were used to detect statistically significant differences between study groups, using StatSoft's Statistica software. Fischer Exact test was applied on non-parametric dichotomous variables. P values below 0.05 were considered statistically significant, and P values below 0.001 were considered highly significant.

## Results

### ESBL production among urinary tract pathogens 

A total of 6254 urine samples were collected from patients attending the healthcare facility and were processed for microbial screening. 1362 (21.8 %) samples were positive for bacterial infections while the remaining 4892 were negative and thus not processed further. Out of 1362, 1039 (76.3 %) samples showed *Escherichia coli *infection, 163 (12.0 %) cases were of *Klebsiella pneumoniae, *while the remaining cases corresponded to *Pseudomonas spp., Proteus spp., Enterobacter spp., Acinetobacter spp., Enterococcus spp., Staphylococcus spp. *and *Streptococcus spp. *(Table 1(Tab. 1)). Among 1039 *E. coli *isolates, ESBL producing isolates were 484 (46.6 %) while the negative isolates for ESBL production were 555 (53.4 %). ESBL producing *K. pneumoniae *isolates were 40/163 (24.5 %) while the *K. pneumoniae *isolates negative for ESBL production were 123/163 (75.4 %).

### Higher UTI prevalence in female population

**Figure 1:**
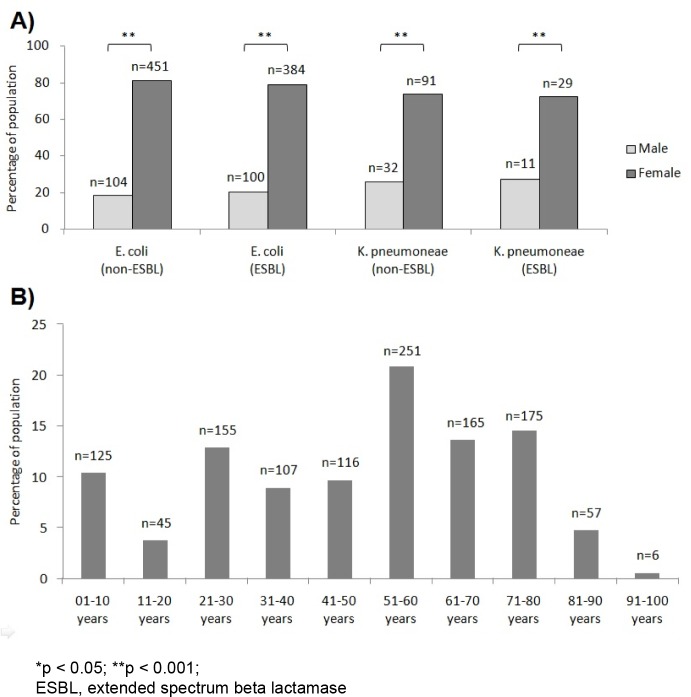
A) Gender distribution of ESBL and non-ESBL producing *E. coli* and *K. pneumoniae* isolates causing urinary tract infections. B) Prevalence of urinary tract infections among various age groups.

Among the patients suffering from infection by non-ESBL *E. coli *isolates*, *104/555 (18.7 %) were males and 451/555 (81.3 %) were females (Figure 1A(Fig. 1)). Among the ESBL producing isolates of *E. coli*, 100/484 (20.7 %) were infecting males while 384/484 (79.3 %) were infecting females. Regarding *K. pneumoniae, *32/123 (26 %) patients affected by non-ESBL *K. pneumoniae* were males while 91/123 (74 %) were females. ESBL producing *K. pneumoniae *affected 11/40 (27.5 %) males and 29/40 (72.5 %) females. Overall, 247/1202 (20.5 %) patients having UTI were males while the proportion of females was significatly higher i.e. 955/1202 (79.5 %). 

### Age distribution of patients suffering from urinary tract infections

Patients suffering from urinary tract infections due to bacterial pathogens belonged to all age groups. Most of the patients (251/1202; 20.9 %) reporting UTIs were between 51-60 years of age. 14.6 % patients were 71-80 years old, 13.7 % patients were 61-70 years old and 12.9 % patients were 21-30 years of age. It is notable that prevalence of UTIs was also quite high in children aged 01-10 years as 10.4 % patients belonged to this age group. The details are given in Figure 1B(Fig. 1). 

### Antibiotic susceptibility testing of Escherichia coli isolates 

**Figure 2:**
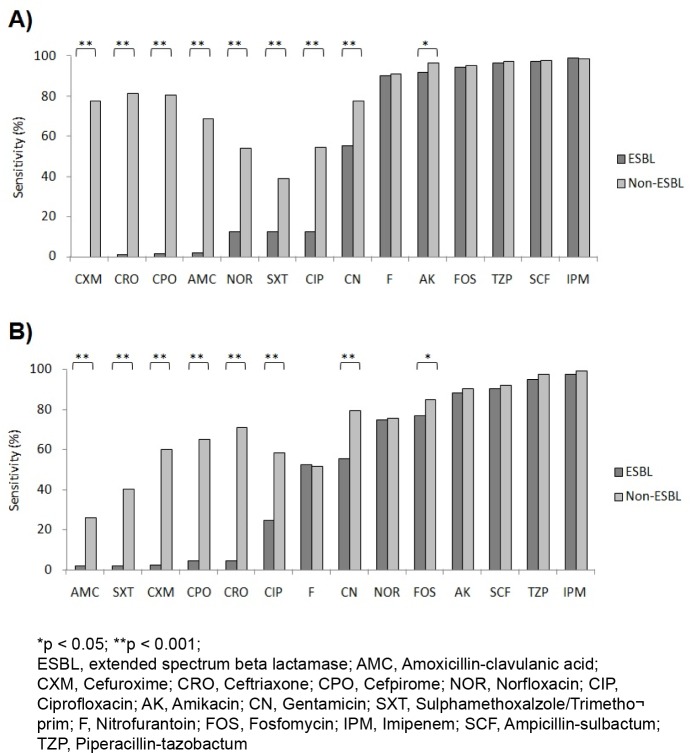
A) Antibiotic susceptibility patterns of ESBL and non-ESBL producing *E. coli* isolates against routinely used antibiotics. B) Antibiotic susceptibility patterns of ESBL and non-ESBL producing *K. pneumoniae* isolates against routinely used antibiotics.

The ESBL producing bacteria were found to be more resistant towards various antibiotics as compared to non-ESBL pathogens (Figures 2A and 2B(Fig. 2); Table 2(Tab. 2)). The most effective drugs found in susceptibility testing against *E. coli *ESBL isolates were imipenem (99.54 %), ampicillin-sulbactam (97.48 %), piperacillin-tazobactam (96.86 %), fosfomycin (94.51 %), amikacin (92.26 %) and nitrofurantoin (90.68 %). The non-ESBL producing isolates were also most susceptible to the same antibiotics (Table 2(Tab. 2)). Significant difference in susceptibility between ESBL and non-ESBL isolates was found in case of amoxycillin clavulanic acid, sulphamethoxalzole/trimethoprim, cefuroxime, cefpirome, ceftriaxone, ciprofloxacin and norfloxacin. The ESBL isolates of *E. coli* were extremely resistant to these set of antibiotics but on the contrary, non-ESBL isolates showed considerable sensitivity to them. The details are given in Figure 2A(Fig. 2) and Table 2(Tab. 2). 

### Antibiotic susceptibility testing of Klebsiella pneumoniae isolates 

The most effective drugs found in susceptibility testing against *K. pneumoniae *ESBL isolates were imipenem (97.62 %), piperacillin-tazobactam (95.35 %), ampicillin-sulbactam (90.48 %) and amikacin (88.37 %). The non-ESBL producing isolates were also most susceptible to the same antibiotics (Figure 2B(Fig. 2), Table 2(Tab. 2)). Significant difference in susceptibility between ESBL and non-ESBL isolates was found in case of beta lactam drugs amoxycillin clavulanic acid, sulphamethoxalzole/trimethoprim, cefuroxime, cefpirome, ceftriaxone and ciprofloxacin. The ESBL isolates of *K. pneumoniae *were highly resistant to these set of antibiotics; however, non-ESBL isolates showed relatively higher sensitivity to them. The details are given in Figure 2B(Fig. 2) and Table 2(Tab. 2).

## Discussion

The most common type of urinary tract infection is the infection of bladder also known as cystitis. The major cause of cystitis is *E. coli* which is a type of bacteria commonly found in the gastrointestinal tract. It was observed that the prevalence of UTIs was considerably high in females as compared to males. One potential reason for cystitis is cross infection through sexual intercourse but women are generally more prone to UTIs because of their anatomy as distance from the urethra to the anus is short and so is the distance from the urethral opening to the bladder. All the age groups were affected by bacterial pathogens but majority of the population belonged to 50-60 years of age. Young children were also affected by ESBL producing *E. coli* and *K. pneumoniae*. Presence of such resistant strains in children is alarming and is potentially linked with the prevalence of such strains in the surrounding people and environment (Fan et al.*,* 2014[11]). 

Antibiotic resistance is an important public heath concern around the globe. Beta lactam antibiotics are the most widely used antibiotics and contain a beta-lactam ring in their molecular structure and usually work by inhibiting bacterial cell wall biosynthesis. Often, bacterial pathogens develop resistance to beta-lactam antibiotics through the production of a beta-lactamase enzyme that attacks the beta-lactam ring and renders the beta-lactam antibiotics ineffective. According to the present findings, the drugs effective against ESBL producing isolates were essentially effective against the non-ESBL producers as well. However, various drugs effective against non-ESBL producers did not work against ESBL-producing pathogens pointing towards the resistance posed by ESBL production. 

The current study reports the overall prevalence of ESBL producing *E. coli* to be 46.6 % and *K. pneumoniae* to be 24.5 %. The ESBL production values are very high and in line with studies being done in Iran (67 %), Latin America (44.9 %), Bangladesh (43 %), India (42 %), United Arab Emirates (41 %), Kuwait (31.7 %), Greece (27.4 %), Bahrain (22.6 %) and Portugal (15.5 %). However, in some studies the prevalence of ESBL producing pathogens was as low as 6.3 % (Saudi Arabia), 2.6 % (Germany) and 2 % (Netherlands) (Winokur et al.*,* 2001[29]; Bouchillon et al.*,* 2004[5]; Rahman et al.*,* 2004[19]; Al-Zarouni et al., 2008[2]; Mehrgan and Rahbar, 2008[15]; Mokaddas et al.*,* 2008[17]; Taneja et al.*,* 2008[26]; Bindayna et al.*,* 2009[4]). The comparatively low rates of ESBLs reflect appropriate use of antibiotics and effective implementation of infection control measures to control the spread of these strains.

Carbapenems work by the inhibition of cell wall synthesis and are highly resistant to the beta-lactamase enzyme. The representative of this class, Imipenem was the most effective antibiotic against non-ESBL as well as ESBL-producing *E. coli and K. pneumoniae *according to the present findings. This is in line with the previous reports from India and Pakistan (Babypadmini and Appalaraju, 2004[3]; Akram et al.*,* 2007[1]; Ullah et al.*,* 2009[28]). Next in line for the effective management of UTIs were the penicillins given in combination with additional cell wall inhibitors e.g. Ampicillin-sulbactam and Piperacillin-tozobactum. These combinations are more effective as both cohorts attack bacterial cell wall; one by interfering with synthesis and other by enhancing degradation and thus rendering bacteria ineffective. These were again very potent against non-ESBL as well as ESBL-producing *E. coli* and *K. pneumoniae*. Piperacillin-tozobactum combination remains at the top (Sabir et al.*,* 2014[21]) while selecting drug of choice and in order to keep it on the long favorable track, this drug must be carefully advised and administered. Ampicillin-sulbactam; however, has shown better results as compared to a study in Ontario, Canada where its effectiveness was only 81 % (Zhanel et al.*,* 2000[30]). This may be attributed to lesser use of this antibiotic in this part of the world. It is important to mention that beta-lactam penicillins like ampicillin, piperacillin, amoxicillin etc. have beta-lactam ring and the ESBL-producers easily overcome their effect so they must be given in combination to guarantee their effect.

Among amino glycosides, Amikacin has shown a good activity against clinically important gram negative bacilli as also reported in earlier studies (Winokur et al., 2001[29]). The susceptibility of ESBL-producing *E. coli* against Amikacin was better than ESBL-producing *K. pneumoniae*. Amikacin generally works through inhibition of bacterial protein synthesis by binding to 30s ribosome leading to misreading of mRNA. Based on the present findings, amikacin is a likely alternative for empirical therapy when other agents cannot be used, but there are no clinical data published on mono-therapy with this agent that would confidently support this argument.

Based on ESBL production, contrary to *Klebsiella pneumoniae*, *Escherichia coli* isolates were highly susceptible to fosfomycin which is similar to the findings of Falagas et al. (2010[10]) suggesting that the *E. coli* have not yet developed any defense mechanism against this drug and it could be used as empirical choice of treatment. However, it was not much effective against *K. pneumoniae* isolates as the resistance reached up to 24 %. 

Nitrofurantoin is a member of Nitrofurans group of antibiotics and renders its bactericidal activity through the damage of bacterial DNA. This antibiotic was effective against ESBL and non-ESBL producing *E. coli* but quite ineffective against ESBL and non-ESBL-producing *K. pneumoniae* isolates suggesting their selection only for the former cases. The sensitivity reported by some previous studies was as high as 99.5 % but this value has now dropped to 90.7 % but still this drug could be used as first line choice to treat urinary tract infections caused by *Escherichia coli *(Zhanel et al.*,* 2000[30]).

The Cephalosporins are beta-lactam antibiotics that disrupt the synthesis of the peptidoglycan layer of bacterial cell walls and thus compromise their structural integrity. However, the ESBL-producing *E.coli* and *K. pneumoniae* isolates showed up to 95 % resistance to the cephalosporin representatives used in this study like cefuroxime, ceftriaxone and cefpirome. All these members have shown much reduced susceptibility as compared to previous studies which may be attributed to the development of the potent resistance mechanism involving the production of beta-lactamase enzyme (Shah et al., 2002[22]). 

Amoxicillin-clavulanic acid was found to be least potent against the ESBL-producing *E. coli *and *K. pneumoniae*. Amoxicillin is a beta-lactam antibiotic so because of the emergence of ESBL production phenomenon, it is given along with clavulanic acid which is a beta-lactamase inhibitor. However, despite giving in combination with clavulanic acid, such low efficacy points to some additional resistance mechanism unidentified so far. Some previous studies also showed compromised efficiency of augmentin but more than 95 % resistance suggests complete elimination of this drug from clinical practice (Rodríguez-Baño et al., 2004[20]).

Sulfamethoxalzole/Trimethoprim is a bactericidal which hinders the folic acid synthesis in the bacterial cell and thus attacks the metabolism. This combination was again least potent against ESBL as well as non-ESBL producing *E. coli* and *K. pneumoniae*. Some previous studies also showed similar findings and suggested to stop practicing the use of this antibiotic (Ullah et al., 2009[28]). Extensive use of this antibiotic may have led to this level of resistance. By now, this drug cannot be suggested as an empirical therapy to treat UTIs in Pakistan.

Among the non β-lactams, gentamicin showed higher affectivity against the bacterial isolates as compared to Israel, India and some other regions of Pakistan (Tankhiwale et al.*,* 2004[27]; Colodner et al.*,* 2007[9]; Ullah et al.*,* 2009[28]). On the contrary, norfloxacin and ciprofloxacin, which are 2^nd^ generation fluoroquinolones and work as DNA synthesis inhibitors, showed reduced effectiveness than reported by earlier studies which may be attributed to increased resistance with time due to misuse of antibiotics and thus supports the hypothesis of this study (Shah et al., 2002[22]). 

To summarize, this study shows that larger number of *E. coli *recovered from UTI in this region produce ESBLs. Thus, they are resistant to penicillins and cephalosporins, which are important drugs in the treatment of UTIs. Such isolates are also resistant to fluoroquinolones, aminoglycosides and tetracyclines. Carbapenems are the drugs of choice against UTIs caused by *E. coli and K. pneumoniae*. The higher MDR in this region is a cause of concern. The findings suggest persistent increasing trend of antibiotic resistance and proportion of ESBLs production by the organism. This indicates the need to focus on regulatory affairs for constant surveillance, proper antibiotic administration and application of stringent infection control processes in order to decrease ESBL frequency. Also, further molecular studies are recommended to elucidate the basis of this multidrug resistance and ESBL production.

In conclusion, the fraction of ESBL-producing isolates is increasing with time that shows higher resistance to a wide variety of commonly used antibiotics as compared to the non-ESBL-producers. The antibiotics showing greater susceptibility towards *E. coli* and *K. pneumoniae* isolates are imipenem, piperacillin-tazobactam, ampicillin-sulbactam and amikacin. The antibiotics having the highest resistance, particularly against the ESBL producers were amoxycillin clavulanic acid, sulphamethoxalzole/trimethoprim, cefuroxime, cefpirome, ceftriaxone and ciprofloxacin and should be removed from the line of treatment for common urinary tract infections. It is strongly recommended to follow the 'Good Clinical Practices' and not to prescribe drugs without appropriate lab tests. Proper counseling of patients should be mandatory for proper and complete courses of medications to avoid the evolution of resistant strains.

## Acknowledgements

The authors acknowledge University Research Fund (URF), Quaid-iAzam University, Islamabad, Pakistan for financial assistance of this study.

## Conflict of interest

The authors declare that they have no conflict of interest.

## Figures and Tables

**Table 1:**
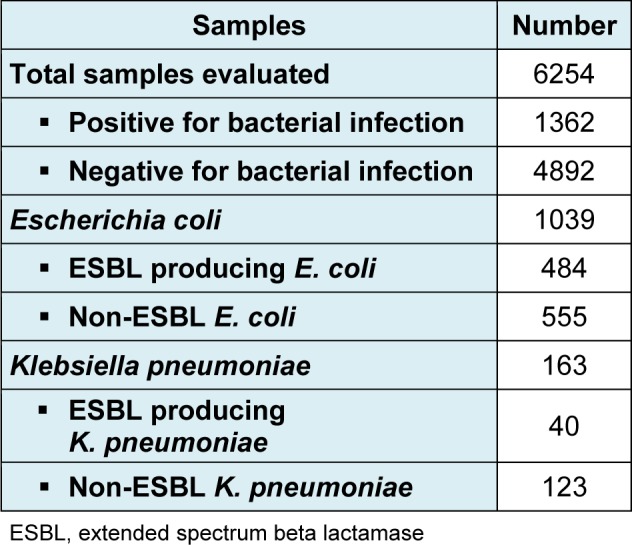
Prevalence of ESBL and non-ESBL producing *E. coli* and *K. pneumoniae* among urinary isolates

**Table 2:**
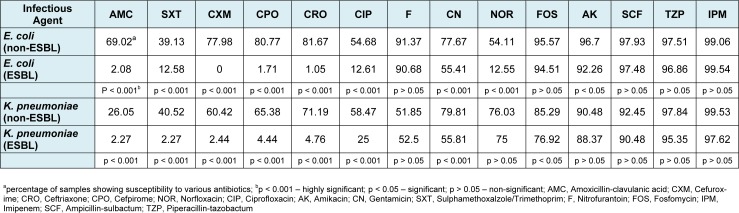
Susceptibility of ESBL and non-ESBL producing *E. coli* and *K. pneumoniae* to routinely used antibiotics
